# Increased resting-state functional connectivity in suprasellar tumor patients with postoperative visual improvement

**DOI:** 10.7150/ijms.35660

**Published:** 2019-08-14

**Authors:** Jianyou Ying, Chuzhong Li, Taoyang Yuan, Lu Jin, Rui Wang, Zhentao Zuo, Yazhuo Zhang

**Affiliations:** 1 Beijing Neurosurgical Institute, Capital Medical University, Beijing, China; 2 Department of Neurosurgery, Beijing Tiantan Hospital, Capital Medical University, Beijing, China; 3 State Key Laboratory of Brain and Cognitive Science, Institute of Biophysics, Chinese Academy of Sciences, Beijing, China; 4 Sino-Danish College, University of Chinese Academy of Sciences, Beijing, China; 5 CAS Center for Excellence in Brain Science and Intelligence Technology, Chinese Academy of Sciences, Beijing, China; 6 Beijing Institute for Brain Disorders Brain Tumor Center, Beijing, China; 7 China National Clinical Research Center for Neurological Diseases, Beijing, China

**Keywords:** suprasellar tumor, visual impairment score (VIS), resting-state functional magnetic resonance imaging (rs-fMRI), local functional correlation (LCOR), functional connectivity (FC).

## Abstract

**Background and Objective:** Large suprasellar tumors often compress the optic chiasm and give rise to visual impairment. Most patients have significantly improved visual function at 1 to 4 months after chiasmal decompression surgery, and only a few individuals regain normal vision at 1 week after surgery. How the recovery of visual function in these patients affects the visual cortex is not fully understood. In this study, we aimed to investigate alterations in brain functional connectivity (FC) in suprasellar tumor patients with visual improvement using resting-state functional magnetic resonance imaging (rs-fMRI).

**Methods:** This longitudinal study was conducted on 13 suprasellar tumor patients who had ophthalmological examinations and rs-fMRI at the following time points: within 1-week preoperation (Pre-op), 1-week postoperation (Post-1w) and 1-month postoperation (Post-1m). The visual impairment score (VIS), local functional correlation (LCOR) and FC values were subjected to one-way ANOVA. Pearson correlation coefficients between changes in the LCOR and clinical factors were calculated.

**Results:** The VIS was significantly decreased at both Post-1w and Post-1m compared to that at Pre-op. Whole-brain analysis of LCOR values showed that the left V1 (primary occipital cortex) was increased significantly at Post-1m compared to that at Pre-op (p < 0.05, FDR corrected). ROI analysis exhibited a significant negative correlation between the LCOR and VIS changes at Post-1m compared to those at Pre-op (p < 0.05, r = - 0.60). FC analysis within the visual network showed that the FC strengths were significantly increased between the left V5 and the left V4, right V3a, left V3, left V2d, and right V5 at Post-1m compared to those at Pre-op (p < 0.05, FDR corrected). Additionally, the FC strengths were significantly increased between the left V5 and the left V1, right orbital-frontal gyrus and left posterior supramarginal gyrus at the whole-brain network level at Post-1m compared to those at Pre-op (p < 0.05, FDR corrected).

**Conclusions:** Postoperative visual improvement can be reflected by the increased FC of the visual cortex at Post-1w and Post-1m, especially at Post-1m. The LCOR value of the left V1 was associated with improved visual outcomes and may be used to objectively assess early visual recovery after chiasmal decompression at Post-1m.

## Introduction

Large suprasellar tumors often compress the optic chiasm and give rise to the most common manifestations of progressively decreasing visual acuity or bitemporal hemianopsia 1. Most suprasellar tumor patients exhibit improved vision function after chiasmal decompression by excision of the lesions 2. Many of these patients have significantly improved visual function at 1 to 4 months after surgery, and only a few individuals regain normal vision at 1 week after surgery 3. The subjective assessment of visual function with ophthalmological examination requires cooperation of the participant and is susceptible to various factors 4. Little is known about how the visual cortex is affected by the recovery of visual function after chiasmal decompression in these patients.

Resting-state functional magnetic resonance imaging (rs-fMRI) plays a vital role in exploring functional interactions between spatially distinct brain regions that reflect the spontaneous fluctuations in brain activity associated with intrinsic behavior 5, 6. The functional integration of brain networks has been evaluated by data-driven functional connectivity (FC) and local functional correlation (LCOR) analysis 7. FC analysis was used to investigate significant functional alterations of the brain by examining the temporal correlations between different brain regions using different statistical methods 8. LCOR, defined as the amount of FC between a voxel and an adjacent voxel across the whole brain in a binary network, may indicate that the voxel in question plays an important role in information processing 9, 10. Numerous fMRI studies have shown that the visual cortex undergoes FC changes following peripheral damage to the visual system 11-15. Task-based fMRI and rs-fMRI studies on pituitary adenoma patients have elucidated that neural dysfunction or FC changes in the visual cortex coincide with partial vision impairment due to chiasmal decompression 16-20. These studies either transversely focus on preoperative neural activity and FC related to visual impairment or longitudinally concentrate on postoperative neural activity changes in the visual cortex. To our knowledge, there has been no longitudinal rs-fMRI study using LCOR and FC in the vision-related cortex to chart the visual recovery after chiasmal decompression in suprasellar tumor patients.

Taking this approach, thirteen suprasellar tumor patients with visual improvement after chiasmal decompression at the 1-week postoperation (Post-1w) and 1-month postoperation (Post-1m) time points compared to that within 1-week of the preoperation (Pre-op) were recruited. We attempted to longitudinally investigate alterations of the FC and LCOR of the visual cortex with visual improvement in 13 suprasellar tumor patients using rs-fMRI in the context of chiasmal decompression and to correlate the fMRI findings with the clinical visual statuses of the patients. We hypothesized that the FC within the vision-related cortex would increase with visual improvement after surgery, especially at the Post-1m time point.

## Materials and methods

### Participants

We initially included 52 suprasellar tumor patients who presented with visual impairment due to lesion-induced compression of the optic chiasm from June 2018 to December 2018. Ultimately, thirteen of these patients with complete data (including ophthalmological examinations and rs-fMRI at the Pre-op, Post-1w and Post-1m timepoints) and postoperative visual recovery at Post-1w and Post-1m were recruited. No patients received adjuvant radiotherapy during the Post-1m period. Participants were recruited according to the following exclusion criteria: left-handed; visual impairment related to corneal opacity, glaucoma, cataract, fundus lesions, optic neuropathy, myopia ≥ -6.00D, hyperopia ≥ +6.00D or other ophthalmologic problems confirmed by ophthalmologic examination; history of stroke, cerebral trauma or other intracranial space-occupying lesions; neurological or mental disorders; history of diabetes, coronary artery disease or other severe illness; no addictions to alcohol or heroin; and inability to undergo MRI and neuroophthalmological examinations.

The Institutional Review Board of Beijing Tiantan Hospital affiliated with Capital Medical University approved the procedures used in the present study. Each participant signed a written informed consent form after understanding our research objectives. Our experimental procedures were based on relevant regulations and guidelines.

### Visual assessment

All patients underwent ophthalmological evaluation at 3 time points. An independent ophthalmologist assessed the visual field, measured the visual acuity and performed fundoscopy. The visual acuity and visual field were separately assessed using Snellen's chart and a standardized automated perimeter (Octopus900 Perimetry, Switzerland). We combined visual field and visual acuity measurements to calculate visual impairment scores (VISs) based on guidelines of the German Ophthalmological Society as reported previously 21, 22. For example, one patient had a visual acuity of 0.2 in the left eye and 0.4 in the right eye together with a bitemporal visual field defect. According to the guidelines of the German Ophthalmological Society, visual acuity is represented by the number 35, and visual field defects are represented by the number 22. Twenty-two plus thirty-five equals fifty-seven, and this sum of 57 represents the VIS, which ranges from 0 to 100. Higher VISs indicate worse visual function and vice versa.

### Image acquisition

The experiment was carried out on a 3T Siemens Prisma MRI scanner (Siemens Healthineers, Erlangen, Germany) using a commercial 64-channel head coil at Beijing Neurosurgical Institute. Each individual high-resolution structural MR image was acquired through a three-dimensional sagittal magnetization-prepared rapid acquisition gradient-echo sequence (224 slices; TI/TE/TR = 1000/2.22/2400 ms; flip angle = 8°; bandwidth = 220 Hz/px; data matrix = 320 × 300; field of view = 256 × 240 mm^2^ with a resolution of 0.8 mm isotropic voxels). Two expert radiologists examined the possible lesions in the cortex using structural images for all participants as exclusion criteria. The rs-fMR images were obtained with an echo-planar image sequence (72 slices; TE = 30 ms; TR = 710 ms; flip angle = 54°; bandwidth = 2358 Hz/px; data matrix = 106 × 106; multiband factor = 8; field of view = 212 × 212 mm^2^ with a resolution of 2.0 mm isotropic voxels) 23. During a 7-min and 41-s functional scan, patients were required to relax, not think of anything and gaze at a fixation point in the central screen throughout the session. After the scan, each subject was asked if he/she remained awake during the whole procedure.

### Data preprocessing

The preprocessing of rs-fMRI data was conducted with Statistical Parametric Mapping (SPM12, http://www.fil.ion.ucl.ac.uk/spm) and the CONN toolbox 24. The first ten volumes were removed to avoid initial signal instability. The preprocessing steps comprised head motion correction, slice timing correction, spatial normalization to the standard Montreal Neurological Institute (MNI) brain space (2 mm), and spatial smoothing (6 mm full width half maximum Gaussian kernel). To eliminate physiological high-frequency cardiac and respiratory noise and reduce low-frequency drift, a temporal bandpass (0.01-0.1 Hz) was performed. Linear trend removal within each time series was also carried out. The head motion, global brain signal, white matter signal and cerebrospinal fluid (CSF) signal were regressed out from the time course of rs-fMRI.

### LCOR analysis

LCOR maps characterized local brain coherence by integrating the spatial Pearson correlation function for each voxel. LCOR is characterized by the strength and sign of connectivity between a given voxel and the neighboring areas in the brain. The FC strength was measured based on the Pearson correlation coefficient of the time courses between the current and neighboring voxels. LCOR is defined as the average of correlation coefficients between each individual voxel and a region (kernel size was 6 mm) of neighboring voxels 9.

### Relationship between LCOR and clinical factors

Pearson correlation analysis between changes in LCOR values in the vision-related cortex and clinical factors (VIS and duration) was performed to determine whether the LCOR varied with clinical factors. Significance was determined using p < 0.05.

### Seed-based resting-state functional connectivity (RSFC) analysis

To calculate the RSFC, we used ROIs as seeds to assess correlations between adjacent brain regions. In this study, 16 nodes (including 8 nodes in each brain hemisphere, V1, V2v, V2d , V3a, V3, VP, V4, and V5; their MNI coordinates, see Table 1) with a 5 mm radius were defined as ROIs to calculate the ROI-wise correlation matrix in the visual networks 25. The coordinates of these selected nodes were acquired according to a previous study 26. First, all clusters were extracted from the corrected correlation map in the standard MNI space. Then, we calculated the Pearson correlation coefficients between the average time courses for every seed region and then converted to z values by Fisher's z transformation. Finally, the brain regions with significantly different FC to the seed regions were confirmed by two-sample paired t-tests between the preoperative and Post-1m periods. Significance was determined using *p*FDR (false discovery rate) < 0.05 at the cluster level and* p* < 0.001 at the voxel level.

## Results

### Demographic and clinical factors

**Figure 1:**
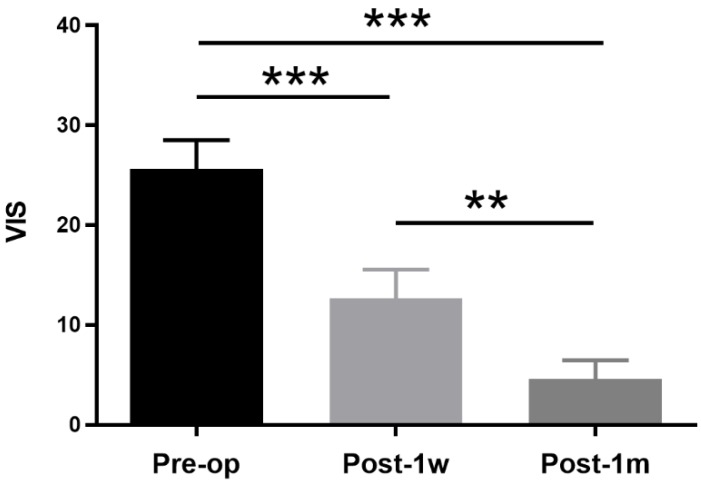
** VISs at Pre-op, Post-1w and Post-1m. The VIS gradually decreased after surgery.** A significant treatment effect was observed from Pre-op to Post-1w (t(12) = 5.14, p < 0.001), and a significant recovery effect was observed from Post-1w to Post-1m (t(12) = 4.68, *p* < 0.002) (** p<0.01, *** p<0.001).

The demographic and clinical features of the suprasellar tumor patients are shown in Table 2. A total of 13 participants were recruited, including 8 (61.54%) female patients and 5 (38.46%) male patients. The age ranged from 34 years to 58 years with a mean age of 46.46±6.86 years. The duration ranged from 1 month to 36 months with a mean time of 8.47±10.33 months. Eleven of the suprasellar tumors were pituitary adenomas, and two were meningiomas. All lesions extended upward to the sella, and 6 also extended to the cavernous sinus. The vision functions of all patients with preoperative partial visual impairment were improved via the transsphenoidal approach except for one, who was treated with the transcranial approach. The VIS decreased at both Post-1w and Post-1m in all patients compared to that at Pre-op. One-way ANOVA was performed among the 3 time points, and a significant main effect was observed(F(2,24) = 48.09, *p* < 0.0001). Tukey's multiple comparisons test was then performed. A paired t-test showed significant treatment effects between Pre-op (25.62 ± 10.44) and Post-1w (12.69 ± 10.27) (t(12) = 5.14, p < 0.001) and significant recovery effects between Post-1w and Post-1m (4.62 ± 6.70) (t(12) = 4.68, *p* < 0.002) (see Figure 1).

### Local functional correlation analysis

**Figure 2:**
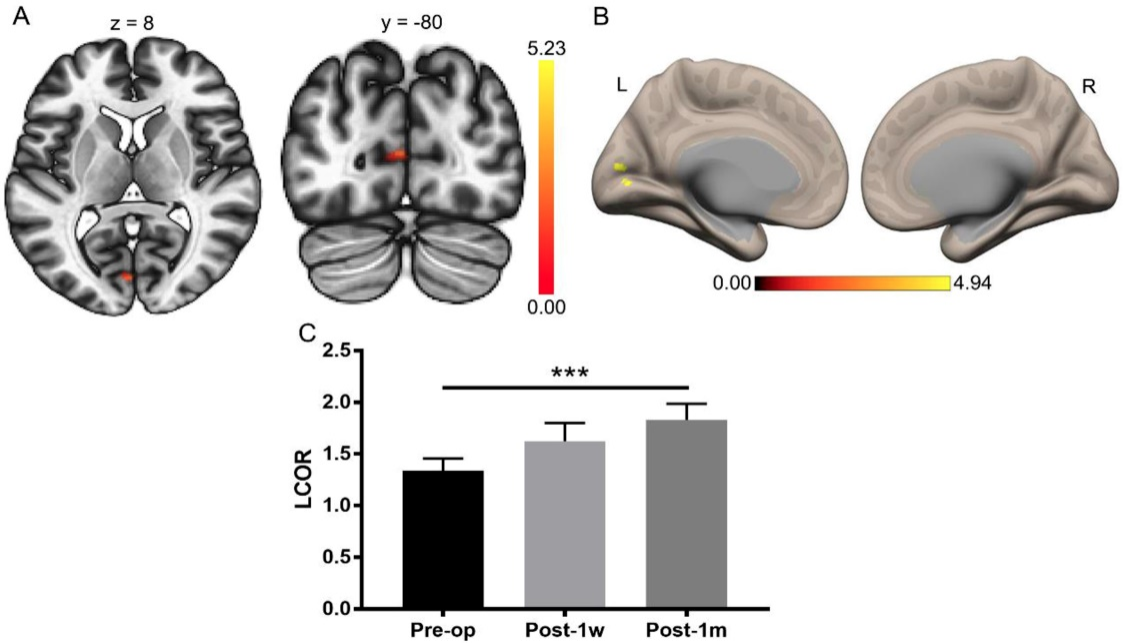
** Whole-brain LCOR analysis of Pre-op and Post-1m. (A)** Statistical parametric map (axial and coronal view). **(B)** Surface brains (3D view). **(C)** The LCOR values of the left V1 at Post-1m are significantly higher than those at Pre-op (t(12) = -6.07, p< 0.001) (*** p<0.001). L, left; R, right.

The visual function improved substantially after Post-1m, as previously reported 3. A paired t-test was applied to the whole-brain LCOR analysis of Pre-op and Post-1m with a cluster-level threshold of *p*FDR < 0.05 and a voxel-level threshold of *p* < 0.001. The only significant difference was found in the left V1 (primary visual cortex) with center MNI coordinates (-2, -82, 10), and the cluster size was 44 voxels. The LCOR values were extracted from the left V1 across the Pre-op, Post-1w, and Post-1m time points (see Table 3). One-way ANOVA was performed among the Pre-op, Post-1w, and Post-1m groups, and the main effect was significant (F(2,24) = 9.15, *p* < 0.005). Tukey's multiple comparisons test was then performed, revealing significantly higher LCOR values in the left V1 at Post-1m than at Pre-op (see Table 3 and Figure 2). There were no significant differences between the other groups.

### Correlation analysis

**Figure 3:**
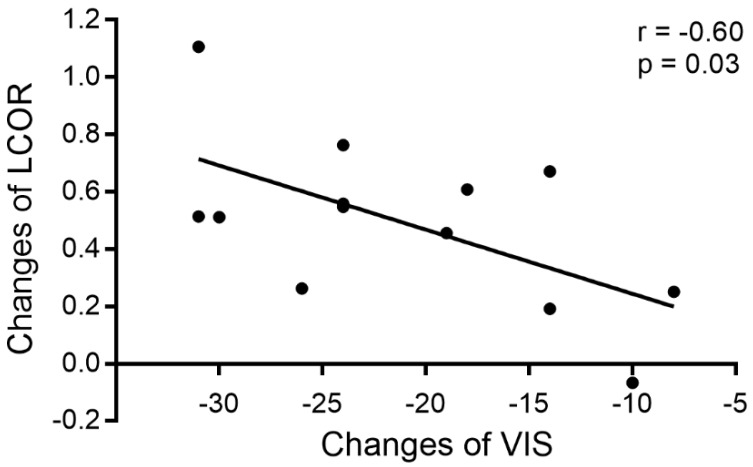
Correlation between changes in LCOR values in the left V1 and changes in VIS from the Pre-op period to the Post-1m period.

Correlation analysis of changes in LCOR values in the vision-related cortex and clinical factors (VIS and duration) was performed at the 3 time points, revealing that the increased LCOR values in the left V1 were associated with decreased VISs after surgery. The changes in LCOR values in the left V1 and VIS from the Pre-op period to the Post-1m period were significantly negatively correlated (r = -0.60, -0.8652 to -0.07405, *p* < 0.05, see Figure 3). No other significant correlations were observed.

### Visual network analysis

**Figure 4:**
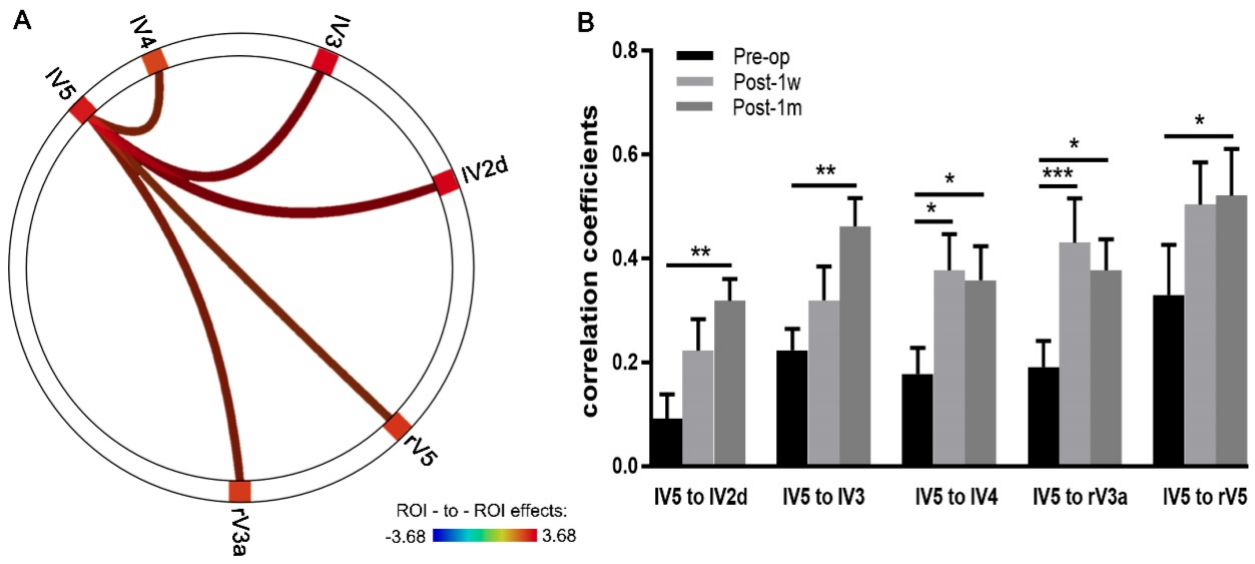
** Depictions of FC strength improved in the visual networks after surgery. (A)** Circle plots depict the visual network with significantly increased FC between the Pre-op and Post-1m period (the left V5 and the left V2d, right V3a, left V3, left V4 and right V5). **(B)** Coefficients of five edges at the Pre-op, Post-1w and Post-1m time points (* p<0.05, ** p<0.01, *** p<0.001). l, left; r, right.

ROI-to-ROI Pearson correlation analyses within the visual network across a time series were conducted using CONN. Multiple comparison-corrected paired t-tests were performed between the Pre-op and Post-1m groups. The correlation coefficients of FC significantly increased between the left V5 and the left V2d, right V3a, left V3, left V4 and right V5 (see Table 4 and Figure 4A).

The correlation coefficients from the above five edges with significant changes in FC were extracted, and two-way ANOVA of 3 time points × 5 edges was performed. Significant effects across Pre-op, Post-1w and Post-1m (F(2,24) = 11.03, *p* < 0.0005) and main effects for edges (F(4,48) = 3.32, *p* < 0.02) were observed. However, no significant interaction effect was found (F(8,96) = 0.82, *p* = 0.59).

One-way ANOVAs were performed on each edge. For the FC strength of edges between the left V5 and left V2d, left V3, right V3a, and left V4, significant main effects were observed across the 3 time points (Geisser-Greenhouse corrected, see Table 5). Tukey's multiple comparisons test was then performed. Paired t-tests showed significant effects on the edges of left V5 and left V2d, left V5 and left V3, left V5 and right V3a, left V5 and left V4 and left V5 and right V5 between Pre-op and Post-1m and on the edges of left V5 and right V3a and left V5 and left V4 between Pre-op and Post-1w (see Figure 4B and Table 5).

### Whole-brain FC analysis based on left V5

**Figure 5:**
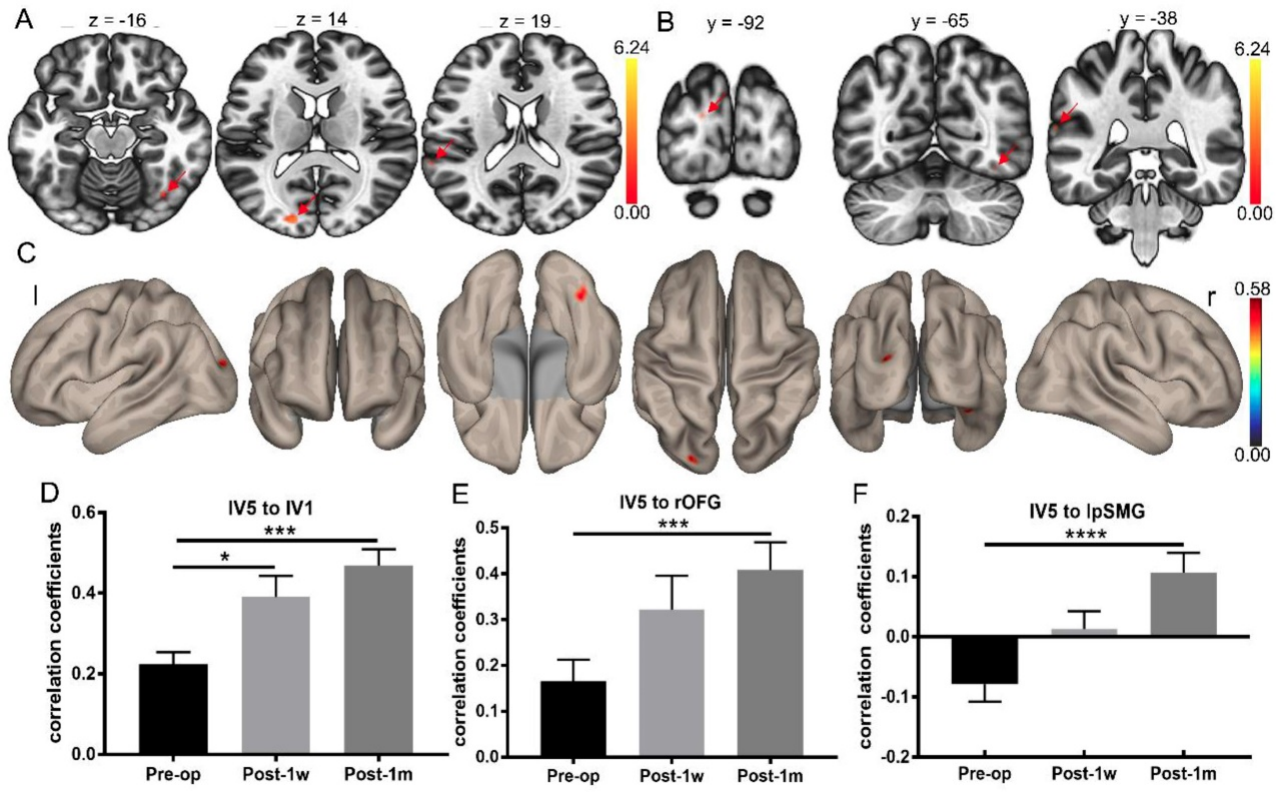
** Whole-brain FC strength alterations after surgery determined using the left V5 as a seed. (A)** Statistical parametric map between Pre-op and Post-1m (axial view, arrows point to the significantly different regions). **(B)** Statistical parametric map between Pre-op and Post-1m (coronal view, arrows point to the significantly different regions). **(C)** Surface brain between Pre-op and Post-1m (3D view). Coefficients between the left V5 and left V1 (D), rOFG (E) and lpSMG (F) at Pre-op, Post-1w and Post-1m (* p<0.05, *** p<0.001). l: left; r: right.

Whole-brain FC analysis was conducted using left V5 as the seed. Significant FC changes were observed between Pre-op and Post-1m using *p*FDR < 0.05 at the cluster level and *p* < 0.001 at the voxel level as the thresholds. The FC map of the left V5 was significantly different regarding connectivity with the left V1, right orbital-frontal gyrus and left posterior supramarginal gyrus regions (see Table 6 and Figure 5A-C).

For FC between the left V5 and left V1 (Geisser-Greenhouse corrected F(1.78,21.32) = 12.96, *p* < 0.0003), rOFG (Geisser-Greenhouse corrected F(1.75,20.97) = 10.85, *p* < 0.001) and lpSMG (Geisser-Greenhouse corrected F(1.48,17.82) = 13.41, *p* < 0.0007), significant main effects were observed. Tukey's multiple comparisons test was then performed. Paired t-tests showed significant treatment effects in the FC of the left V5 and left V1, left V5 and rOFG and left V5 and lpSMG between Pre-op and Post-1m and in the FC of the left V5 and left V1 between Pre-op and Post-1w (see Figure 5D-F and Table 7).

## Discussion

The current study explored FC changes in the vision-related cortex in suprasellar tumor patients with postoperative visual improvement at the voxel level, ROI level and whole-brain network level based on rs-fMRI. The LCOR values significantly increased in the left V1 at Post-1m compared to those at Pre-op, and LCOR changes were negatively correlated with VIS changes from Pre-op to Post-1m. The FC strength significantly increased between the left V5 and other brain regions at both the ROI level and the whole-brain network level. The patients with visual improvement after chiasmal decompression exhibited improved FC in the vision-related cortex at Post-1w and Post-1m, especially at the Post-1m.

The regional homogeneity (ReHo) values of the occipital cortex were reduced in pituitary adenoma patients compared with those in healthy controls 20. In addition, the ReHo values were increased when comparing postoperative pituitary adenoma patients with visual improvement with preoperative patients 19. However, no direct evidence has shown the recovery effects of suprasellar tumor patients after chiasm decompression. In this study, 13 suprasellar tumor patients with visual improvement after surgery were included, and longitudinal comparisons between the Pre-op and Post-1w and Post-1m time points were conducted. LCOR values from the left V1 were increased after surgery at Post-1w and Post-1m compared with those at Pre-op, while the differences were significant at only Post-1m and not at Post-1w. V1 is subjected to internal and external factors and varies with different visual statuses 27. Pituitary adenoma tumors reduce the signals from the retina into the visual cortex, which may reduce the activity of V1 [18, 20]and the local FC within the V1 region, causing blurred images from visual input. Decompression of the optic chiasm allowed more visual stimuli to be received from the retina, as determined based on the observation of increased visual cortex activity and the rebuilding of FC with adjacent neurons. However, the connection was not so stable at Post-1w, and some patients thus did not gain vision improvement at this time point; these patients might need a longer recovery time 16, 17, such as 1-4 months.

Although the VISs of the patients we included in this study were significantly decreased after chiasmal decompression, the LCOR values in the left V1 were significantly higher than those at Pre-op at only Post-1m and not at Post-1w. It seems that the visual function occurred before the visual network recovered. The VIS measures and subjectively assesses the deficit of visual function 21, 22. After chiasmal decompression, retinal signals transmitted to the visual cortex could increase as the visual field improves. However, the local FC may still be unstable, as some studies have shown that visual acuity improvement lags behind that of the visual field 28-31, while others have shown that improvement of the visual field lags behind that of visual acuity 32, 33. One study on pituitary adenoma patients showed that visual function was significantly improved at 1 to 4 months after surgery, and only a few individuals regained normal vision at 1 week after surgery 3. In this study, we found that changes in the LCOR values in the left V1 lagged behind visual changes, which might mean that changes in the LCOR were caused by increased visual information stimulation. The brain will change its FC to improve the efficiency of information processing so that its function can adapt to the current needs 34-36. Many fMRI studies have documented the involvement of V1 in visual learning, which can increase neural activity and FC with trained stimuli 37-40.

Hyperconnectivity was a common response to neurological disruption in patients with multiple sclerosis, traumatic brain injury, idiopathic generalized epilepsy, mild cognitive impairment, and Alzheimer's disease 41-44. Some hyperconnectivity may exist within the visual cortex after surgery in pituitary adenoma patients. Previous studies demonstrated that the ReHo and FC of the V1 region in pituitary adenoma patients were significantly decreased compared to those in the healthy control group 18, 20.

In our study, the LCOR values in the left V1 gradually increased after chiasmal decompression in the process of visual recovery, and the LCOR values in the left V1 were significantly increased at only the Post-1m compared to those at Pre-op. The LOCR values at Post-1m were not higher than those at Post-1w, and those at Post-1w were not higher than those at Pre-op. Therefore, we believe that the increase in FC strength after surgery was a functional recovery mechanism of the visual cortex through connecting adjacent neurons rather than hyperconnectivity within the visual network. Changes in the LCOR value were correlated with the recovery of vision function, as described by the VIS.

Located at the top of the ascending branch of the middle temporal sulcus, V5 (MT+) is a visual area specialized for motion perception in the visual modality 45, 46. The left V5 is activated when a moving object is imaged, but that in the right hemisphere is not 47. In the present study, visual network analysis showed that the positive FC values between the left V5 and the left V2d, left V3, left V4, right V3a and right V5 were increased at Post-1m compared to those at Pre-op in suprasellar tumor patients with visual improvement. When the LCOR values increase, the details of the image or object become sharper, which leads to enhanced sensitivity of motion perception. The left V5 increased the FC with the dorsal stream of the visual cortex, which improved the “where” pathway.

More interestingly, whole-brain FC analysis using left V5 as the seed revealed that the FC map of the left V5 with the left V1, right orbital-frontal gyrus and left posterior supramarginal gyrus was significantly altered before and after the operation. These regions beyond the visual cortex are involved in the processes of language and sensation 48-50, and the reason for the alterations in FC are unknown. We speculated that these patients might exhibit increased communicative ability with vision recovery after surgery, and the connection among higher-order cognitive networks would then be strengthened, as mentioned earlier in association with the fact that the brain increases its FC to adapt to information processing needs 34-36.

There are some limitations in the present study. First, the small sample sizes constituted the main limitation of this study, which might reduce the reliability of the findings. Second, a control sample was not included in the study, and having a control sample would be beneficial for clarifying FC changes before and after surgery. Third, we explored the FC changes with only rs-fMRI, and other neural changes associated with improved vision might be determinable using voxel-based morphometry (VBM), diffusion tensor imaging (DTI) and task-based fMRI analyses. Multimodal neuroimaging analysis may be the direction for investigating the mechanisms underlying structural and functional alterations.

## Conclusion

In summary, postoperative visual improvement can be reflected in the increased FC of the visual cortex at Post-1w and Post-1m, especially at Post-1m. The increased FC of the vision-related cortex might indicate that the rehabilitation course changed in accordance with improved vision input. The LCOR of the left V1 was associated with improved visual outcomes and may be used to objectively assess early visual recovery after chiasmal decompression at the Post-1m time point. Our study may provide important insights into functional alterations related to the vision-related cortices of suprasellar tumor patients with visual improvement.

## Figures and Tables

**Table 1:** MNI coordinates of ROI-wise seed nodes.

Region name	Peak MNI coordinate		
	x	y	z
V1.L	-6	-87	2
V2v.L	-7	-79	-5
V2d.L	-11	-96	8
VP.L	-14	-76	-8
V3.L	-18	-93	12
V3a.L	-26	-87	17
V4.L	-22	-70	-8
V5.L	-41	-75	3
V1.R	8	-83	5
V2v.R	10	-74	-1
V2d.R	10	-91	11
VP.R	15	-73	6
V3.R	15	-91	16
V3a.R	20	-86	21
V4.R	22	-77	-11
V5.R	46	-66	-1

L: left; R: right.

**Table 2:** Demographic data and clinical factors of all patients.

	n	Gender	Age (years)	Duration (months)	Handedness	Approach	Pathology	VIS		
								Pre-op	Post-1w	Post-1m
Patients	13	8F / 5M	46.5±6.9	8.5±10.3	right	12TS / 1TC	11PA / 2ME	25.6 ± 10.4	12.5 ± 9.8	4.6 ± 6.7

F: female; M: male; TS: transsphenoidal; TC: transcranial; PA: pituitary adenoma; ME: meningioma.

**Table 3:** LCOR values in the left V1 at the Pre-op, Post-1w and Post-1m time points.

Region	Mean (SD)			F(2,24)/p	t(12)/p		
	Pre-op	Post-1w	Post-1m	one-way ANOVA	Pre-op vs Post-1m	Pre-op vs Post-1w	Post-1w vs Post-1m
Left V1	1.34 (0.43)	1.62 (0.64)	1.83 (0.57)	9.15/< 0.005	-6.07/< 0.0002	-2.15/0.121	-1.64/0.265

**Table 4:** Visual network with significantly increased FC between the Post-1m and Pre-op time points.

Network		T	*p*FDR
seed	target		
V5.L	V2d.L	3.68	<0.05
V5.L	V3.L	3.56	<0.05
V5.L	V3a.R	3.08	<0.05
V5.L	V4.L	2.94	<0.05
V5.L	V5.R	2.84	<0.05

L: left; R: right.

**Table 5:** FC strengths of the edges between lV5 and lV2d, lV3, rV3a, lV4 and Rv5 at Pre-op, Post-1w and Post-1m.

Edges	Mean (SD)			F(2,24)/p	t(12)/p		
	Pre-op	Post-1w	Post-1m	one-way ANOVA	Pre-op vs Post-1m	Pre-op vs Post-1w	Post-1w vs Post-1m
lV5 to lV2d	0.09 (0.17)	0.22 (0.22)	0.32 (0.15)	5.48/<0.02	-5.21/<0.01	-2.58/0.20	-1.87/0.41
lV5 to lV3	0.22 (0.15)	0.31 (0.24)	0.46 (0.20)	4.43/<0.05	-5.04/<0.01	-1.90/0.40	-2.02/0.35
lV5 to rV3a	0.19 (0.18)	0.43 (0.31)	0.38 (0.22)	7.11/<0.002	-4.36/<0.05	-5.29/<0.001	1.01/0.76
lV5 to lV4	0.18 (0.18)	0.38 (0.25)	0.36 (0.24)	5.11/<0.02	-4.15/<0.05	-4.24/<0.05	0.36/0.96
lV5 to rV5	0.33 (0.35)	0.50 (0.29)	0.52 (0.32)	3.03/0.08	-5.02/<0.05	-2.38/0.25	-0.29/0.97

**Table 6:** Differences in whole-brain FC determined using the left V5 as the seed at Pre-op and Post-1m.

Region	Coordinate			Cluster size (voxels)	T	*p*FDR
	x	y	z			
Left V1	-18	-90	14	44	7.65	0.008
rOFG	36	-6	-14	34	6.03	0.017
lpSMG,	-66	-38	20	26	5.75	0.042

**Table 7:** FC strengths of edges between lV5 and lV1, rOFG and lpSMG at Pre-op, Post-1w and Post-1m.

Edges	Mean (SD)			F(2,24)/p	t(12)/p		
	Pre-op	Post-1w	Post-1m	one-way ANOVA	Pre-op vs Post-1m	Pre-op vs Post-1w	Post-1w vs Post-1m
lV5 to lV1	0.22 (0.11)	0.39 (0.19)	0.47 (0.15)	12.96/<0.0003	-8.53/<0.0002	-4.21/<0.05	-2.24/0.29
lV5 to rOFG	0.17 (0.17)	0.32 (0.26)	0.41 (0.22)	10.85/<0.001	-8.133/0.0002	-3.71/0.054	-2.21/0.29
lV5 to lpSMG	0.08 (0.10)	0.01 (0.11)	0.11 (0.12)	13.41/<0.0007	-10.82/<0.0001	2.98/0.13	-3.58/0.06
